# Observation of a chemical reaction in a levitating microdroplet cluster and droplet-generated music†

**DOI:** 10.1039/d4sc03066d

**Published:** 2024-06-27

**Authors:** Alexander A. Fedorets, Semyon Koltsov, Anton A. Muravev, Alexey Fotin, Pavel Zun, Nikita Orekhov, Michael Nosonovsky, Ekaterina V. Skorb

**Affiliations:** a X-BIO Institute, University of Tyumen Tyumen 625003 Russia; b ITMO University St. Petersburg 191002 Russia skorb@itmo.ru; c Department of Mechanical Engineering, University of Wisconsin-Milwaukee Milwaukee WI 53211 USA nosonovs@uwm.edu

## Abstract

Ordered clusters of water droplets levitating over a heated water surface can be used as chemical microreactors and computational devices. Here we show that a chemical reaction between melamine and cyanuric acid can occur during coalescence of pairs of droplets containing these reagents and lead to the sedimentation of the product, crystals of melamine cyanurate. In rotating droplets, the crystals flash with frequencies dependent mostly on the rotational velocity of the droplets defined by the rotor of the velocity field of the air–vapor flow above the heated water surface. With the Machine Learning (ML) approach, we trace the brightness and frequencies of individual crystals and convert the frequencies into musical notes thus making a “micro-orchestra” of the levitation cluster.

## Abstract 2

A containerless chemical reaction observed in a levitating droplet cluster for the first time is used for information processing.

## Graphical abstract

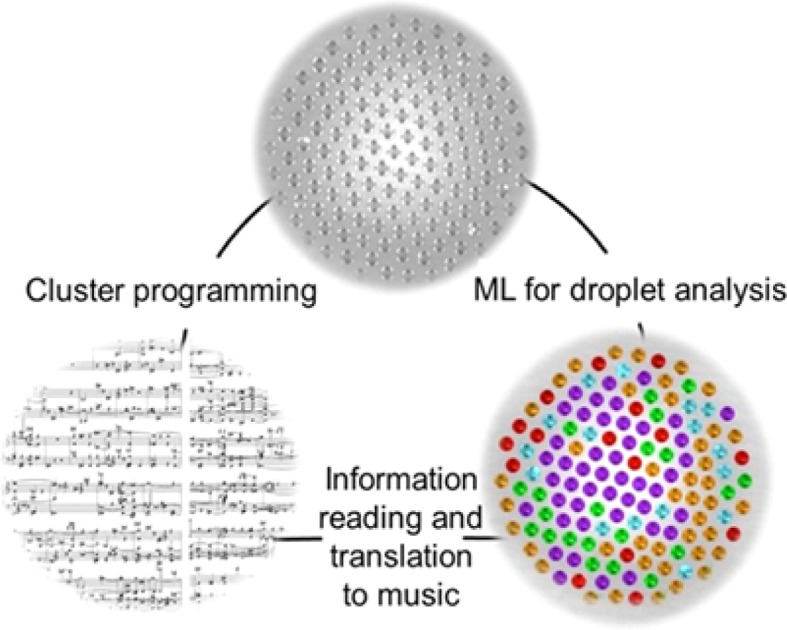

## Introduction

Information processing in colloid systems including particles, microdroplets and microbubbles is a rapidly developing field. In the recent paper of Sitti's group,^1^ the order and information in the pattern generated in a magnetic field are discussed. Cronin and co-workers used a crystallization process as a powerful entropy source for generation of true random numbers^2^ and developed a programmable chemical computer with memory and pattern recognition based on comprising a 5 by 5 array of cells filled with a switchable oscillating chemical (Belousov–Zhabotinsky (BZ)) reaction.^3^ For the chemical computer based on BZ, the chemicals can be placed inside microspheres.^4–7^ Another aspect is the use of microfluidic bubble logic to overcome the limit of the von Neumann bottleneck.^4,5^ The recent advancements in bubble logic computation based on two-phase microfluidics bring into light the possibility that the use of bubbles in microfluidic devices can carry on-chip process control. Thus, all new approaches to run chemical reactions related to computing are interesting as well as the system engineering for these approaches.

Of particular interest are containerless chemical reactions in small droplets.^8^ Acoustic levitation of droplets has been studied for at least two decades. Matsubara and Takemura^9^ showed that an acoustically levitated floating droplet has potential as a chemical and biological reactor. These are droplets in the range of 1 to 10 μL with a typical linear size on the order of 10^−3^ m. They note that containerless processing could lead to a next-generation reactor without risks of unforeseen issues induced by contact with the container, such as alteration and contamination of the reactants.^9^

Self-assembled clusters of condensed microdroplets levitating over a locally heated water surface were reported for the first time by Fedorets in 2004 (ref. ^10^) and have been intensively studied after that.^11–16^ When a thin (about 1 mm) water layer is heated locally to the temperatures at which water evaporates actively (40–95 °C), small water droplets (5 μm to 50 μm in radius) are condensed in the ascending flow of mixed air and water vapor. Microdroplets are of particular interest due to their high surface-to-volume ratios and potential catalytic effect of the free surface. These droplets form monolayer clusters levitating at heights comparable with their radii. Such clusters are typically self-assembled into an ordered hexagonal structure due to an interplay of aerodynamic forces, which drag the droplets towards the center of the heated flow facilitating closed (hexagonal) packing, and aerodynamic repulsion forces between droplets facilitating a distance between them.^11,12^ Small clusters may possess symmetries absent from the large clusters or colloid crystals including the 4-fold, 5-fold, and 7-fold symmetries,^13,14^ while large clusters demonstrate phase transitions between hexagonal, chain-like,^15^ and hierarchical arrangements.^16^

Since droplets in clusters can be stabilized for a significant time length (hours),^17^ they can serve as a tool to study processes in aerosol droplets, such as the bacteria life cycle, which are very difficult to observe in free flying droplets. It has been hypothesized that droplets in a cluster can be used as chemical and biological microreactors and even as chemical information processing/computing devices.^14^ For the latter, logic gates should be implemented, for which the coalescence of two droplets, containing solutions of different substances, or droplet division, may be used.

The melamine cyanurate complex (M–CA) has been studied for more than a decade due to its unique structural properties and due to its potential for programmable assembly of functional materials.^18,19^ Every M molecule can connect to three CA molecules by 9 strong N–H⋯O and N–H⋯N hydrogen bonds, and *vice versa*, to form a 2D supramolecular network of C_3_H_6_N_6_ melamine (M) and C_3_H_3_N_3_O_3_ cyanuric acid (CA) molecules.^19^ Both components can form three H-bonds directed at an angle of 120° and located in the same plane, which makes them perfect counterparts to each other.^20^ When the two components meet, the high energy of the intermolecular interactions between them leads to the instant precipitation of M–CA from solution in the form of a fine crystalline powder. The recrystallization and grain coarsening are not observed due to negligible solubility of the resulting compound. Crystalline nucleation is a relatively slow stage in the M–CA precipitate formation, while their growth, although limited by the diffusion of the reagents to the particle's surface, proceeds much faster.

We show here that a chemical reaction between melamine (M) and cyanuric acid (CA) can be realized in the levitating microdroplet cluster during coalescence of pairs of droplets containing water solutions of these reagents. Such a reaction leads to the sedimentation of the product, crystals of melamine cyanurate (M–CA). The M–CA system is governed by strong hydrogen bonding and soft π–π aromatic stacking interactions constituting an example of molecular self-assembly. We will use a random generation of crystals in a levitating droplet cluster as an information-carrying entity for studying the relationship between information and the dynamic behavior of the system. Moreover, we will transform information through musical notes. For such a system, it is done for the first time.

## Results

### Observation of M–CA crystals

**Fig. 1:**
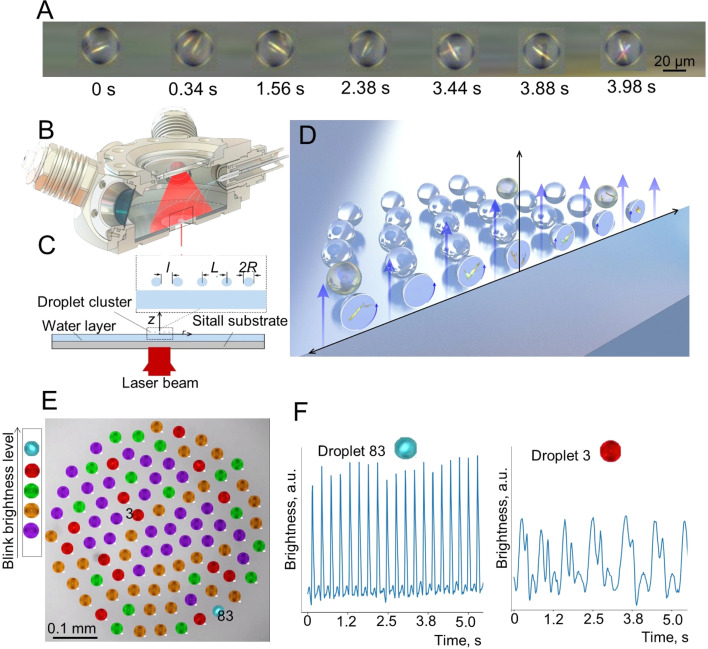
Observation of chemical reactions in a levitating droplet cluster. (A) M–CA crystal in a rotating droplet, frames selected for the timesteps when the crystal was best seen. Relative time shown. (B) The schematic of a reactor to inject solutions of M and of CA. (C) The side view of the reactor. (D) Schematic of droplets in an ascending air-vapor flow. Only vertical component of the drag force is shown. (E) Droplet cluster analysis using the machine learning (ML) approach showing different crystals in them. Blue, red, green, orange and purple colors were assigned automatically and represent droplets with different level of blink brightness of periodically oscillating crystals, aperiodic oscillations, chaotic light flickering, and no crystals, respectively. (F) Oscillating brightness of rotating droplets shown for two droplets, #3 and #83. Droplet #3 has lower frequency since it is located closer to the center of the cluster. See Videos S1 and S2 of cluster behavior in time with and without ML clustering in the ESI.†

20 mM solution of CA and 20 mM solution of M were injected into the reactor as microdroplet clouds using an ultrasound nebulizer. Some of the droplets containing the M solution merged with droplets containing the CA solution due to coalescence triggering the M + CA → M–CA reaction between the two substances. After a short (1–2 s) period of chaotic motion, many droplets disappeared and the cluster stabilized forming a self-assembled ordered structure of almost monodisperse droplets. The M–CA crystals could be observed visually inside some droplets of the clusters. Since the droplets rotated, the needle-like or leaf-like M–CA crystals often appeared periodically in the droplets as reflected light flashes. These flashes lasted typically for 20–100 ms. The frequency of flickering varied significantly, but typically it was under 4 Hz. Many flashings did not occur regularly. The video of flickering droplets was recorded at 50 frames per second. Fig. 1A shows one droplet containing the M–CA crystal at different time steps selected in such a way that the crystals can be best seen.

The droplet cluster formed in the reactor (Fig. 1B and C) as a self-assembled hexagonal structure due to the aerodynamic drag force towards the center of the cluster where the temperature was at maximum, hence evaporation and ascending gas flow most intensive (Fig. 1D). The droplets levitated at the equilibrium height, comparable with their radii, where their weight was equilibrated by the vertical component of the drag force. In a stable cluster, droplets did not merge due to aerodynamic repulsion force between them.^11^ The gradient of the gas flow velocity resulted in the rotation of the droplets.

Two series of experiments were conducted. In the first series, small droplets (5 μm diameter) were injected with an ultrasonic nebulizer. After an initial period of chaotic motion and condensational growth of the droplets, the cluster was stabilized. About 80–90% of the stabilized droplets contained small crystals. In the second series, the cluster of pure water droplets (30 μm diameter) was first created using an ultrasonic dispenser and then the solutions of the reagents were added using an ultrasonic nebulizer. The goal was to obtain larger clusters than in the first series. Indeed, the crystals in the second series were larger, but the fraction of droplets with crystals was smaller.

Flickering or flashing due to light reflection from the crystal surface was observed in a significant fraction of the droplets that formed the droplet cluster. This fraction varied from about 25% to about 90% among dozens of droplets, although the exact number depended on the experimental conditions. In most cases, only one M–CA crystal was present in every droplet, although in some cases two and even three crystals were observed. M–CA crystals have formed almost instantly after the merging of the M and CA containing droplets. In most cases, the extremity of a needle-like or leaf-like crystal was located at the droplet surface, while the crystal itself was inside the droplet. The typical length of the crystals could be estimated as 10–15 μm, while their thickness and width were 2–5 μm. The diameter of the droplets was 20–50 μm. Due to rotation, droplets with oscillating crystals flashed with reflected light.

A computer program was used to trace individual droplets and to mark their images with different colors depending on whether periodic oscillations were observed (Fig. 1E) as well as to extract the frequencies of oscillations (Fig. 1F). The droplet cluster behavior is seen in Videos S1 and S2.†

### Molecular dynamics (MD) modeling of the effect of the liquid surface

**Fig. 2:**
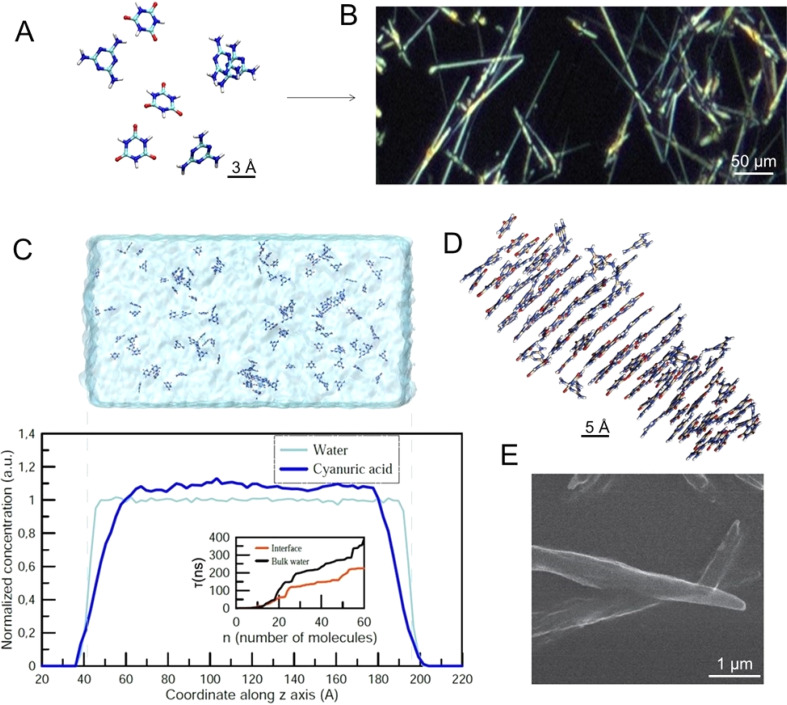
Melamine (M) and cyanuric acid (CA) self-organization. (A and B) Highlight of M–CA crystal growth starting with (A) individual molecules, and (B) final supramolecular crystals formed on glass. (C) Snapshot of the computational cell containing two water–air interfaces and spatial distribution of CA molecules in a computational cell. The inset shows MFPT – the average time *τ*(*n*) required for the largest crystallite in the computational cell to reach the size of n molecules. (D) Needle-like M–CA crystallite containing 75 M and 75 CA molecules obtained by MD modeling. (E) Scanning electron microscopy image of the M–CA crystallite.

The M–CA crystal growth starts with (Fig. 2A) individual molecules forming final supramolecular crystals (Fig. 2B). To estimate a possible effect of the water–air interface on the nucleation and growth of M–CA crystallites we performed a series of molecular dynamics (MD) simulations covering two possible scenarios: nucleation in the bulk water and in the vicinity of the water–air interface. Molecules with hydrophobic chemical groups can have a tendency to accumulate near the surface and, therefore, introduce spatial inhomogeneity in the nucleation process. Such an effect was observed for several amino acids including phenylalanine and methionine in water solution.^21^ As a result, kinetics of chemical and physico-chemical processes can accelerate at the surface of micro-droplets.^22,23^

Following the approach described in our previous paper,^20^ we performed a series of constant-temperature simulations at *T* = 300 K in the computational cell with periodic boundary conditions containing 32 000 water molecules (Fig. 2C). In the “bulk water” scenario, the whole computational cell was filled with water (approximate size 160 × 80 × 80 Å^3^). In the “surface” scenario, water occupied only the central part of the computational cell (approximate size 240 × 80 × 80 Å^3^) and, therefore, two water–air interfaces existed within the cell (Fig. 2C). In all simulations the total number of M and CA molecules was 80 and 80. For each of the two scenarios, we performed five independent simulations with a duration of an individual simulation up to 500 ns.

Fig. 2C shows the space distribution of CA molecules within the computational cell before the nucleation of the crystallite started. One can see that the distribution is homogeneous with a steady decrease in the surface region (approximately 2 nm wide). The inset in Fig. 2C shows the so-called mean first-passage time (MFPT) – the average time *τ*(*n*) required for the largest crystallite in the computational cell to reach the size of n molecules for the first time. The results indicate that nucleation of M–CA crystals only accelerates in the presence of the open surface: in the bulk water, it takes 300–400 ns to form a crystal containing 60 molecules, while for the system with water–air interfaces the same process takes 200–250 ns. The effect presumably results from the increased concentration of M and CA in the central region of the unit cell (due to a decrease in the surface areas – Fig. 2C) and should not significantly influence nucleation processes in the case of a microscopic water droplet. The surface effect was significant only at the distance of about 2–3 nm from the interface. The final structure of the M–CA crystallite formed in the simulation (Fig. 2D) resembles the needle-like shape of crystals experimentally observed in regular macroscopic cuvettes (Fig. 2E).

### Droplet rotation frequency in the droplet cluster

**Fig. 3:**
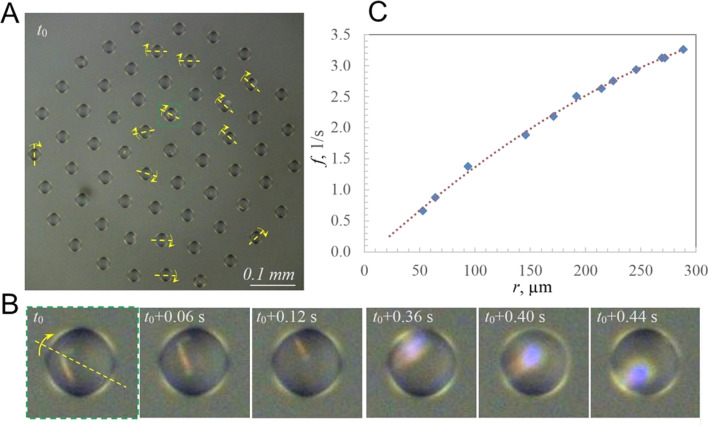
Crystals for the study of droplet rotation dynamics *vs.* position in the cluster. (A) Droplet cluster with the axes of rotation of the droplets containing crystals. (B) A series of snapshots of a droplet at different instances of time showing the crystal when it is in the top part of the droplet (first three snapshots) and a flash when it is at the bottom of the droplet (last three snapshots); (C) the dependency of the frequency of flashes on the distance from the center of the cluster.

As an example, we consider the cluster shown in Fig. 3A, with a zoomed droplet at different time steps shown in Fig. 3B. The droplet contains a significantly large crystal to observe it when it is located at the top of the droplet. During droplet rotation, the crystal remains invisible for some time, and it flashes with reflected light when the crystal is at the bottom of the droplet due to the focusing of the reflected light by the droplet acting as a lens. The time interval between the flashes corresponds to the frequency of the rotation of the droplet (Fig. 3B).

In this cluster, 12 droplets located at different distances from the center had regular flashings. Consequently, the orientation of the axis of droplets' rotation was determined and the dependency of the frequency on the distance from the center was obtained (Fig. 3C). Consistently with the above analysis, the frequency changed from zero at the center to 3.3 s^−1^ at the periphery. The equation of the fit is given by *f* = −0.000017*r*^2^ + 0.016492*r* − 0.117051.

### Temperature control over the size and behavior of the droplet cluster

**Fig. 4:**
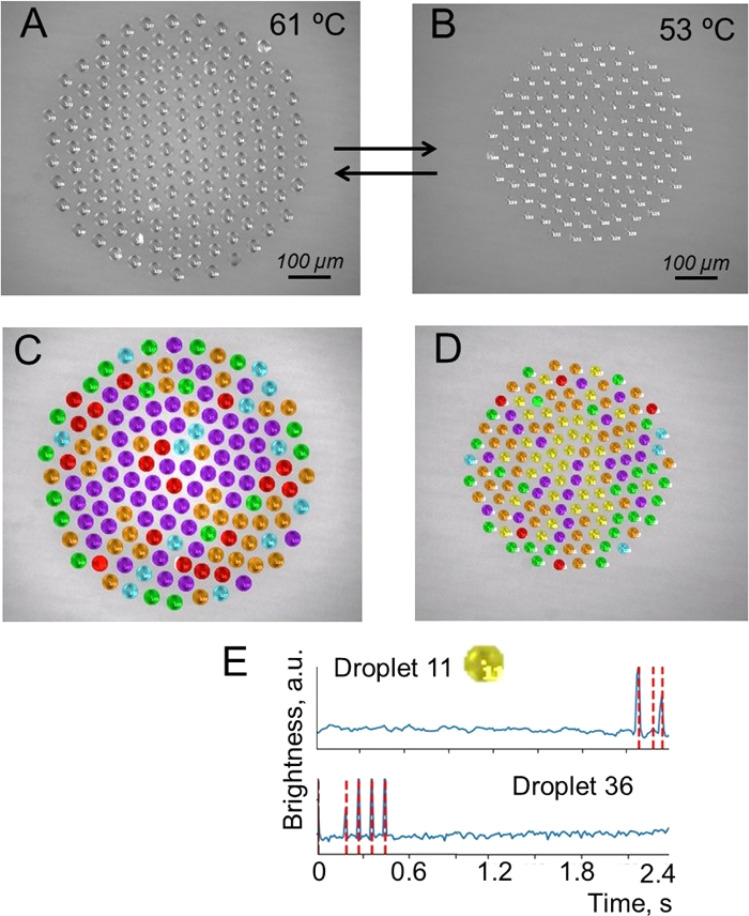
Droplet cluster at different temperatures. (A) Generation of a droplet cluster with 141 droplets in its composition. (B) Change of the droplet cluster size by decreasing the temperature to 53 °C. Note that the decrease of temperature was done slowly with some time for the cluster stabilization not to lose the droplets. (C and D) Droplet cluster analysis using the machine learning (ML) approach. Blue, red, green, orange and purple colors were assigned automatically and represent droplets with two different levels of flashing brightness of periodically oscillating crystals, aperiodic oscillations, chaotic light flickering and no crystals. (E) The sixth new type marked by yellow for the droplet cluster at 53 °C representing rare aperiodic flashes shown for two droplets, #11 and #36. See Videos S3–S6 of cluster behavior in time with and without ML clustering in the ESI.†

In the first series of the experiments, after droplet cluster stabilization, the temperature was slowly decreased from 63 °C to 53 °C. The droplet cluster at 61 °C and 53 °C is shown in Fig. 4A and B, respectively. A computer program was used to trace individual droplets and to mark their images with different colors depending on whether periodic oscillations were observed (Fig. 4C and D). Blue, red, green, orange, purple, and yellow colors were assigned automatically and represent droplets with two different levels of flashing brightness (blue and red) of periodically oscillating crystals, aperiodic oscillations (green), chaotic light flickering (orange), no crystals (purple) and rare aperiodic flashes (yellow). The latter type is marked by yellow for the droplet cluster at 53 °C representing rare aperiodic flashes shown for two droplets, #11 and #36 (Fig. 4E).

This clustering of droplets into six groups based on the periodicity and brightness of blinking worked well for both temperatures (Videos S3–S6†). The brightness of crystal flickering inside the droplet is attributed to reflection from the crystal. While the crystals are not visible to the naked eye, flickering due to reflected light is well seen. At 61 °C, the number of flashing droplets was close to 20% out of a total of 141 droplets. At a lower temperature of 53 °C, just a few droplets without flickering (purple) remained. Close to 80% (blue, red, green, orange and yellow) started to flash. This is likely explained by better observability of the crystals in larger droplets rather than by the formation of new crystals. The oscillation of brightness for “yellow” clustering droplets of #11 and #36 is shown in Fig. 4F.

## Discussion

### Droplet rotation and flashing frequency

Numerous observations of the cluster having droplets with small particles, such as the M–CA crystals and other small particles of similar size, show that these particles tend to rotate together with the droplet as a whole. No considerable relative motion of such particles and no random Brownian motion have been observed, although some aperiodic and chaotic oscillations were found in some droplets. We, therefore, assume that the angular velocity of crystal rotation is close to the angular velocity of the droplet containing the crystal and that the rotation of the droplet is the dominant mechanism for periodic flickering.

Most intensive evaporation of the water layer occurred at the center of the heated area thus resulting in the highest vertical component of the gas stream flow at the center of the heated spot (which is also the center of the cluster). For the vector velocity field *V⃑* = *V*_r_*r̂* + *V*_z_*ẑ* which has the vertical and radial components, *V*_z_ and *V*_r_, the vector of angular velocity can be calculated as a rotor in cylindrical coordinates1
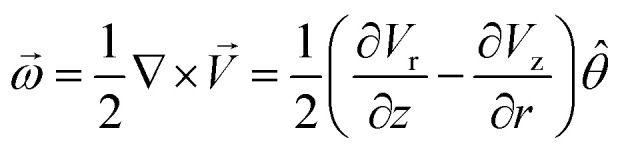
where *r̂*, *ẑ*, and *θ̂* are corresponding orthogonal unit vectors.

The gradient of the vertical component of the velocity 
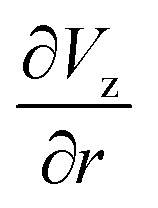
 is zero at the center of the cluster as well as the radial component 
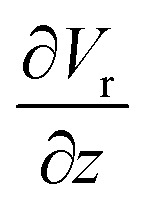
 due to the symmetry of the problem. Therefore, from eqn (1), the rotational velocity *ω*(*r*) = 0 at the center of the cluster. The velocity is also zero at the boundary of the heated spot*ω*(*r*) = 0, where *L* is the radius of the heated spot.

The maximum value of the temperature gradient and therefore of 
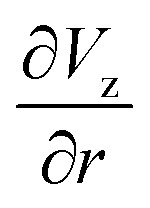
 is achieved between *r* = 0 and *r* = *L*, and it can be estimated as2
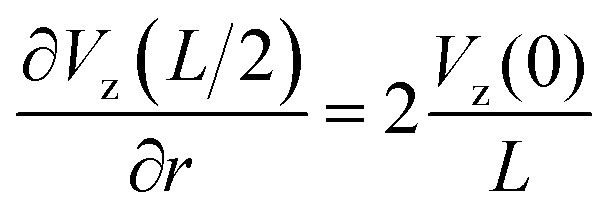


Assuming the typical values *L* = 2 mm and *V*_max_ = 0.05 m s^−1^ and small 
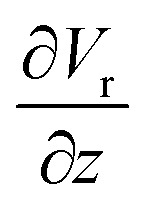
, yields the maximum value of the rotation speed (at *r* = *L*/2 = 1 mm) as *ω* = 25 rad s^−1^ or the frequency of the oscillations 3.98 s^−1^. Note, however, that the cluster is often smaller than *L*/2, and, therefore, the maximum value is not achieved. We see that the observed results on the distribution of the rotational velocity of droplets are consistent with theoretical expectations.

The velocity of the ascending gas flow is dependent on the temperature at the liquid surfaces, and, therefore, by changing the temperature, the frequency of rotation can be controlled.

### Information processing and music generation

We have demonstrated that it is feasible to perform a two-component chemical reaction within the droplets in the cluster. Such reaction can be used for information processing, for example, to implement a logic gate or for the predicates *P*_A_(*N*)*P*_B_(*N*), and *P*_A_(*N*) or *P*_B_(*N*) implying that substances A, B, and “A or B” are contained in the droplet number *N*. While our study demonstrates merging of droplets in a droplet cluster, droplet division by phase separation has also been demonstrated for active droplets.^24^

The sedimented crystals can be used to trace the rotation of the droplets, which is a difficult task otherwise. Moreover, the rotational speeds of the droplets provide the tool to measure the velocity field above the heated water surface. The information about the rotational speeds of the droplets is transformed into the visual mode and then into the sound mode, thus making the cluster a levitation “micro-orchester” generating music.

While close-packed colloidal crystals made of rigid microparticles are a natural phenomenon that has been studied for decades, the droplet cluster has a number of peculiar features, and observing a chemical reaction adds an additional dimension to its study. Cooperative motion, self-assembly of ordered structures, and structural phase transitions in colloidal crystals are similar to effects observed in the condensed matter.^1,25,26^

**Fig. 5:**
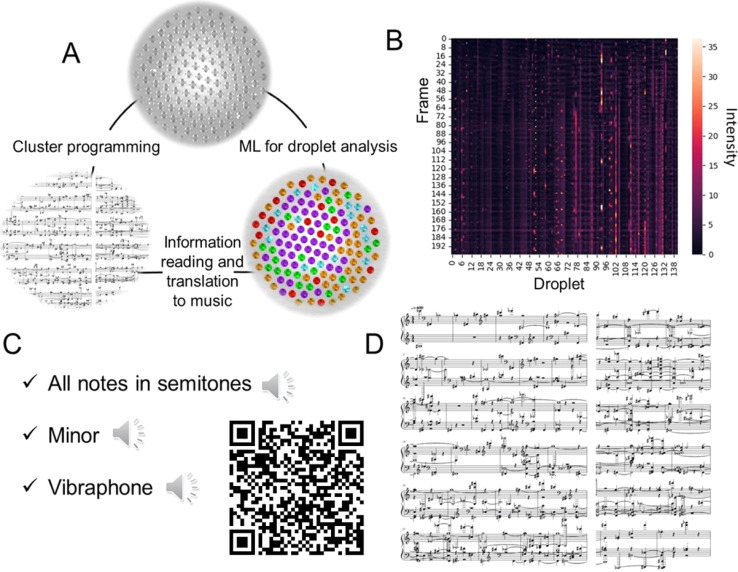
The translation to music of the information from the droplet cluster analysis. (A) The schematic of the information transition from the initial cluster imaging, by machine learning (ML) analysis of brightness change in time and by converting the information to music. (B) Example of frame *vs.* droplet number plot of normalized droplet glow intensity, and (C) musical notes produced from the data in (B). (D) Cluster. Note that different musical notes can be suggested depending on the type of the cluster. Download and listen the corresponding music from the ESI or using the provided QR-code.

In recent literature, music generation is sometimes discussed in the context of signal processing and AI.^27^ To demonstrate the potential of the droplet clusters for information processing, we transformed the information about the flashing droplets into the sound form (Fig. 5A). Each droplet was assigned a musical note. After that, the melody was created. In addition to the frequency of the sound, there are a couple of other important characteristics, the volume and the duration of the sound. The volume is regulated by the normalized intensity of the droplet's glow with a threshold of 15%; if the intensity is too low, the note is not played to avoid background noise. The normalized intensity of each droplet is shown in Fig. 5B. The duration of the sound is controlled by the number of frames in which the intensity of the droplet is above the threshold, preventing duplication of the sound.

The generated MIDI file with the melody can be further converted to .mp3, for example, with the MuseScore^28^ program by selecting any suitable sound sample (ESI S7–S9†). Also, musical notes were created, and arranged for piano for two hands, cluster #3 (ESI S10†). Here, fortepiano samples were used (Fig. 4C). To summarize, the transformation of droplet dynamics into music is an illustrative example of using the microdroplets as an information-carrying entity for studying the relation between information and the dynamic behavior of the system.

## Conclusion

We have observed a chemical reaction between melamine and cyanuric acid in levitating microdroplets of a droplet cluster. First, the cluster of droplets containing a water solution of M was formed by injecting the droplets. After that, droplets of a solution of CA were added, and many of these droplets coalesced including droplets thus triggering a chemical reaction leading to the precipitation of M–CA crystals in some of the droplets. The crystals could be observed directly when at the top part of the droplet and by flashes of reflected light when at the bottom of the droplets due to the lens effect of the droplets. Since droplets rotated with the angular velocity dependent on the gradient of the velocity field of the ascending gas stream, rotating surfaces of the crystals caused periodic flashes with various frequencies. To track droplets and extract frequencies of flashes, a Machine Learning (ML) approach was developed. The information was further converted into sound leading to the droplet cluster-generated music. This work is the first observation of a chemical reaction in the droplet cluster, and it stresses the potential of the cluster for chemical information processing and as an array of chemical microreactors. In our study, the crystallization phenomenon was used for the observation of the chemical reaction. Potentially, it could be used for the droplet logic related to computing.

## Materials and methods

### Experimental

The droplet formed a cluster over a locally heated spot of a horizontal layer of distilled water containing surfactant microadditives (sodium dodecyl sulfate at 0.02 g L^−1^) suppressing thermocapillary flows. The surfactant monolayer at this concentration does not evaporate and does not affect the water microdroplets. The surfactants have no impact on the appearance of melamine-cyanurate self-assemblies, at least at the concentrations below the critical aggregation concentration in the case of sodium laureth sulfate and Triton X-100. A sitall substrate (400 μm thick) was attached to the bottom of the cuvette. The body of the cuvette contained channels connected to the cryothermostat Piccolo 280 OLE (Huber, Germany) allowing temperature stabilization in the water layer (Fig. 1B). Local heating of the water layer was performed with a laser beam (MRL-III-660D-1W, CNI, China) directed towards the lower side of the substrate (Fig. 1C). Small infra-red sources EK-8520 (Helioworks, USA) were used to control the size of the droplets by suppressing the condensational growth of the droplets.

Two series of experiments were conducted. In the first series, 20 mM solution of CA was injected into the reactor as microdroplet clouds created by an ultrasound nebulizer (Omron, Japan), which generated water microdroplets (diameter about 5 μm) by ultrasonic acoustic irradiation. After that, in a similar way, 20 mM solution of M was injected as well. The aerosols were injected when the heating power level was decreased (the local temperature of the water layer at the center of the heating spot was *T*_max_ = 60 ± 2 °C). Some of the droplets containing the M solution merged with droplets containing the CA solution due to coalescence thus forming the solution of the two substances and triggering a reaction between them. After a short (1–2 s) period of chaotic motion, many droplets disappeared and the cluster stabilized forming a self-assembled ordered structure of almost monodisperse droplets. The diameter of the droplets grew from the initial small values. The sedimented crystals of M–CA were observed visually due to light reflected from their surfaces. The laser power was varied to control the droplet temperature and therefore the size of the condensed droplets.

In the second series, at first, the droplet cluster was created with an ultrasound dispenser (MicroFab) with 30 μm diameter droplets. After that, both components, CA and M, were injected through the small (5 μm diameter) droplets. The temperature was first lowered from 56 °C down to 44 °C to check the size of the droplets and for comparison of flashing and not flashing droplets and then further increased up to 65 °C. The third series allowed us to obtain larger crystals.

Video recording at 50 fps was performed with an iXon Ultra 888 fluorescence microscopy camera (Andor, UK); a polarizing filter FS09 was applied in some series of experiments. The temperature of the water surface under the cluster *T*_surf_, was monitored with a CTL-CF1-C3 pyrometric sensor (Micro-Epsilon, USA). In all experiments, the temperature at the periphery of the water layer was maintained at 10 °C by cooling the cuvette with a Piccolo 280 OLÉ cryothermostat (Huber, Germany). It is important to note that even at *T*_surf_ = 65 °C, the humid air flow velocity was sufficient to prevent spontaneously condensing droplets from penetrating into the cluster.

### MD simulations

We performed MD simulations of M and CA assembly using the GROMACS package.^29^ To describe interatomic interactions, we applied the OPLS-AA force field^30^ with partial charges parameterized by LigParGen.^31^ The TIP4P^32^ rigid nonpolarizable model was used to parameterize the water molecules. A cutoff for short-range and nonbonded interactions was 1.2 nm. For long-range Coulomb interactions, we used the smooth Particle-Mesh Ewald scheme.^33^ Visualization was produced using VMD.^34^

### Machine learning analysis of the droplet cluster

The black-and-white video recordings of the cluster were used. Crystals of various sizes and shapes growing in droplets reflect light depending on the position of the crystal in the drop and its size. To trace the droplets and analyze the luminosity of crystals inside them, an automatic analyzer based on artificial neural networks (ANNs) was designed. The tracking algorithm included four stages: (i) searching for the initial position of the droplets, (ii) tracking the position of the droplets, (iii) calculating the droplet glow intensities, and (iv) collecting data. To implement the algorithm, a sliding window ANN was used.

At the first stage of the algorithm, the entire series of frames of a given experiment was scanned. The series was divided into “windows” of a given size, and the ANN processed each “window” to determine whether the object corresponds to the droplet or not. This made it possible to find the centers of all droplets. The second part of the algorithm was to find a new position of the droplets in each subsequent photo in the vicinity of the droplet center of the previous photo. This approach saves a lot of time compared to sliding across the whole picture. A set of coordinates of droplet centers was determined, which, together with the original images, provides a complete description of this experiment. Three types of ANNs were tested: the simplest fully connected, the simplest convolutional and convolutional networks of the U-net type. The results showed that the simplest convolutional network is accurate enough for the algorithm to work. In turn, the use of U-net increases the accuracy of the determination but slows down the analysis process.

The final ANN consisted of three convolutional layers with the MaxPool technique, one fully connected and one classifying layer. The leaky ReLU activation function is used on all layers. The input channel of the original image is one-dimensional. The first convolutional layer has an 8-channel output, the second layer has 16, and the third has 32. Each convolutional layer has 3 × 3-pixel kernels. Batch_norm is used to normalize values by group. The droplet glow intensity was calculated as the sum of all pixels of the square in which the droplet circle is inscribed. Thus, to calculate the intensity of the glow of a drop, we add the values of the pixels that correspond to it, subtract the background value and divide the result by the number of pixels. A combination of K-means clustering and the principal component methods was used, which showed the greatest efficiency. First, the droplet intensity values obtained in the previous step were reduced to a square value by discarding the values until a square of some number was obtained, then the intensity values were written into the matrix to obtain a square black and white image. Next, this graphical representation was extrapolated to a size of 224 × 224 pixels and three identical channels to match the input of the VGG16 neural network. At the output of the neural network, we received 4096 features describing this graph. Feature reduction for clustering was carried out using the principal component method, which made it possible to group droplets with extremely subtle fluctuations.

### Data processing and sound generation

After processing the images as described above, we obtained each droplet's number (sequential ID) and the intensity of its glow over time. In order to generate sounds from the data, a MIDI file was generated for each series of images using the *Midiutil* library for *Python*. In MIDI format, musical notes are numbered from 0 to 127, with C4 (middle C) being number 60. For the clarity of sound and convenience of perception of the generated melody, the whole-tone scale was taken as the basis. Each droplet was assigned a note in the order of numbering, and since the number of drops is greater than the number of selected notes, there is duplication (*e.g.*, droplet #26 is assigned note #1 again).

## Author contributions

Conceptualization: EVS, AAF, and MN. Methodology: EVS, AAF, and MN. Investigation: AAF, AAM, SK, PZ, and AF. Visualization: SK, AF, PZ, and NO. Project administration: EVS and AAF. Supervision: EVS. Writing – original draft: MN. Writing – review & editing: MN, EVS, AAM, and PZ.

## Data availability

All data, code, and materials used in the analysis are available at https://drive.google.com/drive/mobile/folders/1bez359AAb2aGz8cChGh9mlHPHliDooSc.†

## Conflicts of interest

The authors declare that they have no competing interests.

## Supplementary Material

SC-015-D4SC03066D-s001

SC-015-D4SC03066D-s002

SC-015-D4SC03066D-s003

SC-015-D4SC03066D-s004

SC-015-D4SC03066D-s005

SC-015-D4SC03066D-s006

SC-015-D4SC03066D-s007

SC-015-D4SC03066D-s008

SC-015-D4SC03066D-s009

SC-015-D4SC03066D-s010
