# Supplementary material for: Observation of a chemical reaction in a levitating microdroplet cluster and droplet-generated music
Source: Chem Sci. 2024 Jun 27;15(30):12067–76. doi: 10.1039/d4sc03066d (PMC11290446; doi:10.1039/d4sc03066d)

Electronic Supplementary Material (ESI) for Chemical Science.
This journal is © The Royal Society of Chemistry 2024

ITMO University

Sparkling drops

Arranged for piano

"Observation of a chemical reaction in a levitating microdroplet cluster and droplet-generated music"

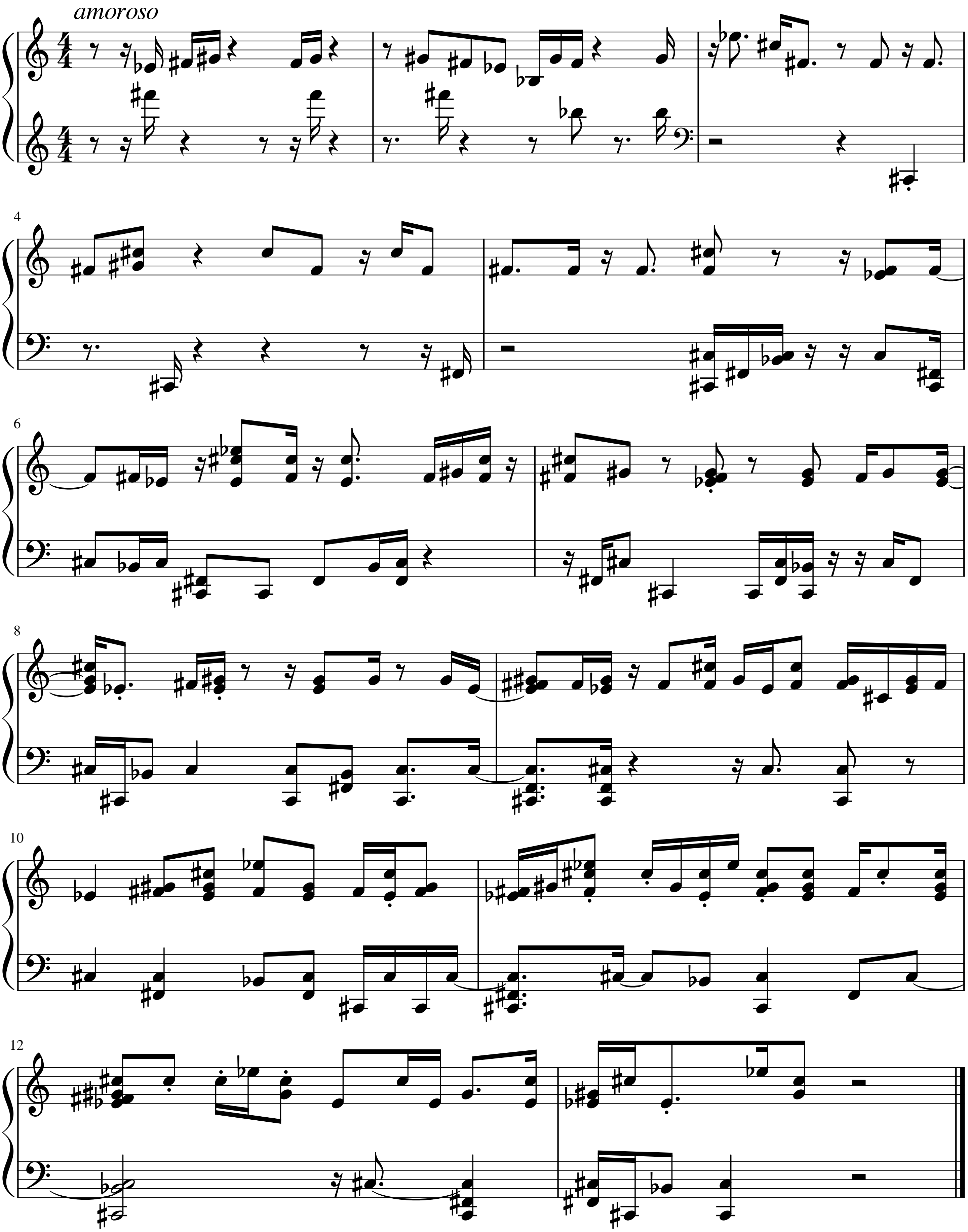

Supplement: SC-015-D4SC03066D-s001 [file SC-015-D4SC03066D-s001.pdf]
